# The Conductive Silver Nanowires Fabricated by Two-beam Laser Direct Writing on the Flexible Sheet

**DOI:** 10.1038/srep41757

**Published:** 2017-02-02

**Authors:** Gui-Cang He, Mei-Ling Zheng, Xian-Zi Dong, Feng Jin, Jie Liu, Xuan-Ming Duan, Zhen-Sheng Zhao

**Affiliations:** 1 Laboratory of Organic NanoPhotonics and CAS Key Laboratory of Bio-Inspired Materials and Interfacial Science, Technical Institute of Physics and Chemistry, Chinese Academy of Sciences, No. 29, Zhongguancun East Road, Beijing, 100190, P. R. China; 2 University of Chinese Academy of Sciences, No. 29, Zhongguancun East Road, Beijing, 100190, P. R. China; 3 Chongqing Institute of Green and Intelligent Technology, Chinese Academy of Sciences, No. 266 Fangzheng Ave, Shuitu technology development zone, Beibei District, Chongqing 400714, P. R. China

## Abstract

Flexible electrically conductive nanowires are now a key component in the fields of flexible devices. The achievement of metal nanowire with good flexibility, conductivity, compact and smooth morphology is recognized as one critical milestone for the flexible devices. In this study, a two-beam laser direct writing system is designed to fabricate AgNW on PET sheet. The minimum width of the AgNW fabricated by this method is 187 ± 34 nm with the height of 84 ± 4 nm. We have investigated the electrical resistance under different voltages and the applicable voltage per meter range is determined to be less than 7.5 × 10^3^ V/m for the fabricated AgNW. The flexibility of the AgNW is very excellent, since the resistance only increases 6.63% even after the stretched bending of 2000 times at such a small bending radius of 1.0 mm. The proposed two–beam laser direct writing is an efficient method to fabricate AgNW on the flexible sheet, which could be applied in flexible micro/nano devices.

Over the past few years, there has been increasing attention on micro/nano devices on flexible substrate due to the unique potential for flexible electronics such as sensory skins for robotics, structural health monitors, wearable communication devices, and so on^1^,^2^,^3^,^4^. Flexible electrically conductive nanowire is now a key component in the fields of flexible devices. Metals are considered as the ideal conductive material, however, metals are rigid and inflexible due to the high Young’s modulus, e.g. 76 GPa in the case of Ag^5^. In particular, the surface morphology of metal nanowire is loose and rough^6^,^7^,^8^,^9^,^10^,^11^, and thus results in the poor conductivity^12^,^13^. Therefore, the fabrication of the metal nanowire with good flexibility, excellent conductivity, compact and smooth morphology is urgently demand for manufacturing the flexible electronic devices.

Precise control of the morphology and optimization of the electrical conductivity of metal nanostructures are the premise for further applications. Much effort has been devoted to achieve the metal nanostructures of controllable morphology and desired conductivity^14^,^15^,^16^,^17^,^18^. A solution-based assembly technique has been utilized to fabricate conductive nanostructures, however, it is difficult to orderly control the morphology of the nanowires^19^,^20^. Conventional imprinting lithography is considered to be a useful method, which is performed at low temperature, low pressure and produces excellent uniformity in the quality of the resulted imprinted structures^21^. Unfortunately, the method requires multi-processes to complete the fabrication, and the samples will be contaminated inevitably. As a material-conserving deposition approach^22^, the inkjet printing technique has been used to fabricate nanowires and devices of different conductive materials on the flexible substrate^23^,^24^. Although the nanowires fabricated by inkjet printing technique exhibit good conductivity, the surface of the nanowires are incompact and rough originating from the uncontrollable size and distance of the metal particles. In our previous work, we have realized the silver nanowire (AgNW)^25^,^26^,^27^ and other metal structures^28^,^29^,^30^,^31^ by femtosecond laser induced multiphoton process on the glass substrate. Nevertheless, the expected continuous and compact AgNW was not achieved from the Ag aqueous solution on the flexible polyethylene terephthalate (PET) sheet only by femtosecond laser, due to the hydrophobic surface of the PET sheet. Therefore, the achievement of compact and smooth metal nanowire with excellent conductivity on the flexible substrate is still a challenge.

In this work, a new two-beam laser direct writing protocol is proposed to fabricate the conductive AgNW with compact and smooth morphology on the flexible PET sheet, owning to the photoreduction of the pulse laser and the optical trapping effect of the continuous-wave (CW) laser. The AgNW with controllable width and height at nanometric scale has been fabricated by optimizing the experimental conditions. We have investigated the electrical resistance under different voltages and the applicable voltage per meter range, which is determined to be less than 7.5 × 10^3^ V/m for the fabricated AgNW. The AgNW electrical resistance dependence on the different bending radii has demonstrated the flexibility. Compared to the AgNW without bending, the resistance hardly changes even after stretched bending of 2000 times at such a small bending radius of 1.0 mm. This proposed protocol for fabricating conductive AgNW of excellent flexibility would open up new avenues for the further application in flexible micro/nano devices.

## Results

### The two-beam laser direct writing system

**Figure 1:**
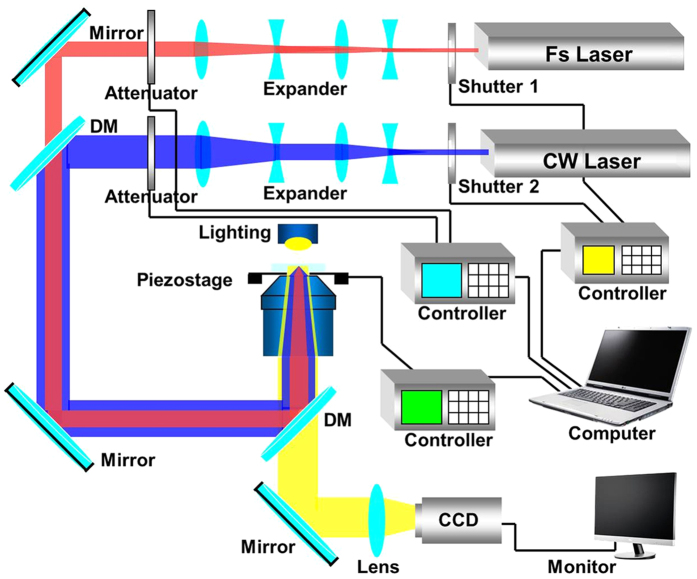
Schematic diagram of the two-beam laser direct writing experiment setup.

The two-beam laser direct writing technique has been employed in creating the continuous and compact AgNW on the PET sheet. A mode-locked Ti:Sapphire laser system (Tsunami, Spectra-Physics) with a center wavelength of 780 nm, a pulse width of 120 fs, and a repetition rate of 80 MHz is used as the pulse laser excitation source, mainly induces the multiphoton photoreduction of Ag nanoparticles. The other CW laser with a wavelength of 442 nm (He-Cd laser, IK57511-G) mainly works as optical tweezer^32^,^33^, which is a promising process to make the Ag nanoparticles gather together by an optical trapping force^34^,^35^,^36^,^37^ and form a continuous and compact AgNW. The two-beam laser direct writing setup is illustrated in Fig. 1. The two beams pass through two laser beam expanders and the diameters of the two beams laser spots are all expanded six times in order to improve the uniformity of the intensity distribution of the laser spot and reduce the laser divergence angle. The beam diameter of pulse laser increases to 6 mm and the beam diameter of CW laser increases to 10 mm. Then the center of the two beams overlapped together by a dichroic mirror (DM) and focused into the sample on the three-dimensional (3D) piezo stage (P-563.3 CD, Physik Instrument) by an oil-immersion high numerical aperture objective lens (Olympus, N.A. = 1.42, ×60). The movement of the piezo stage, shutters and attenuators are controlled by a computer according to a preprogrammed pattern. A CCD camera was mounted after the DM to monitor the fabrication process.

### Schematic illustration of the AgNW preparation strategy

**Figure 2:**
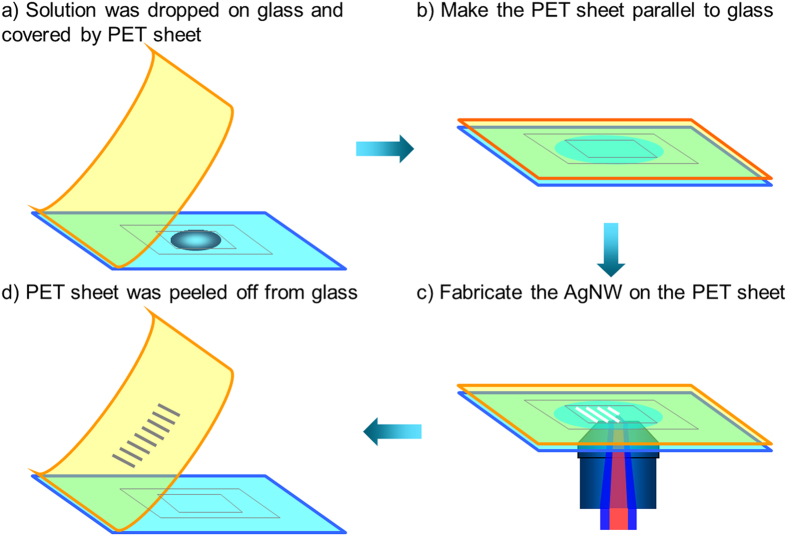
Schematic illustration of preparation of the AgNW on the PET sheet using two-beam laser direct writing. (**a**) Ag aqueous solution is dropped on the glass substrate and covered by PET sheet. (**b**) The sample is sandwiched between the glass substrate and the PET sheet, and the PET sheet is made to be parallel to the glass substrate. (**c**) Two beams are focused into the sample by the objective lens and to fabricate the AgNW on the PET sheet. (**d**) PET sheet is peeled off from glass substrate and the AgNWs are obtained after washing out the unreacted solution with ethanol.

A schematic illustration of the AgNW preparation strategy on flexible sheet is presented in Fig. 2. A hollow film with a thickness of about 50 μm is stuck on a glass substrate as a spacer and thus a home-made cuvette is formed. The sample is filled in the cuvette on the glass substrate (Fig. 2a). The PET sheet is covered above the glass substrate, and the sample is sandwiched between the glass substrate and the PET sheet, forming a space-confined cuvette (Fig. 2b). Then the laser is tightly focused into the inner surface of the PET sheet by the oil-immersion objective lens. The Ag nanoparticles will be photoreduced and the AgNW could be fabricated on the PET sheet with the movement of a piezo stage (Fig. 2c). Finally, the PET sheet is peeled off from the glass substrate and the AgNW is obtained after removing the unreacted solution with ethanol (Fig. 2d).

### Fabricated the AgNW on the PET sheet

We have verified the fabrication capability of both pulse and CW lasers, respectively. The pulse laser itself could only produce some breakpoints of Ag nanoparticles or the very loose AgNW but not the compact AgNW on the PET sheet (Supplementary Fig. S1). On the other hand, some continuous AgNWs can be fabricated on the PET sheet, but the surface is very rough while only using CW laser (Supplementary Fig. S2). Based on the understanding of the effect of two lasers, we try to find out the optimized experimental conditions for fabricating the desired AgNWs on the PET sheet by using two-beam laser direct writing technique.

**Figure 3:**
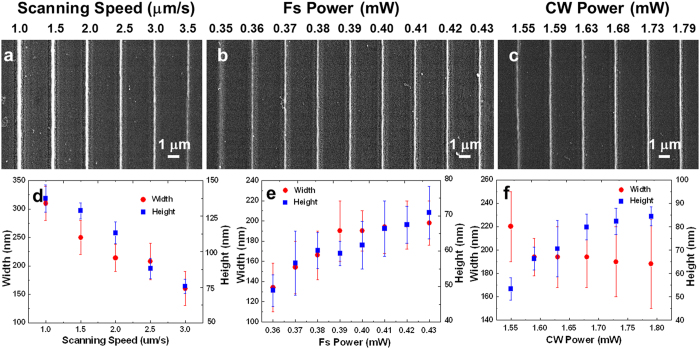
SEM images and the corresponding width and height of the AgNWs on the PET sheet that fabricated under different experimental conditions. (**a**) SEM image of AgNWs fabricated while varying the scanning speed from 1.0 μm/s to 3.5 μm/s. The laser powers of pulse beam and CW beam is 1.79 mW and 0.43 mW, respectively. (**b**) SEM image of AgNWs which were fabricated while the pulse beam power varied from 0.35 mW to 0.43 mW. The CW beam power and scanning speed are 1.79 mW and 3.0 μm/s. (**c**) SEM image of AgNWs which were fabricated under the CW beam power ranging from 1.55 mW to 1.79 mW. The pulse beam power is 0.43 mW and the scanning speed is 3.0 μm/s. (**d**) The dependence of width and height of the AgNWs in (**a**) on the scanning speed. (**e**) The dependence of width and height of the AgNWs in (**b**) on the pulse beam power. (**f**) The dependence of width and height of the AgNWs in (**c**) on the CW beam power.

Scanning speed plays an important role in determining the morphology of the AgNW including width and height. While the laser powers for the pulse beam and the CW beam are kept at 0.43 mW and 1.79 mW, AgNWs are fabricated with different scanning speed. Figure 3a shows the scanning electron microscope (SEM) images of the AgNWs fabricated on the PET sheet with the scanning speed from 1.0 μm/s to 3.5 μm/s. The AgNWs fabricated with a speed less than 3.0 μm/s are continuous, but becomes intermittent when the scanning speed is as fast as 3.5 μm/s. The width and the height of the AgNW are determined by measuring 5 different positions in the SEM images and atomic force microscope (AFM) images (Supplementary Fig. S3a), respectively. Both of the width and the height of the AgNWs decrease with the increasing of the scanning speed (Fig. 3d). It is reasonable that there are not enough Ag nanoparticles to aggregate into a continuous nanowire owing to the decreased amount of the photoreduced Ag nanoparticles when the scanning speed increases to 3.5 μm/s.

The morphology of the AgNW will be influenced by the amount of photoreduced Ag nanoparticles, originating from the multiphoton photoreduction of pulse laser beam. The AgNWs were fabricated on the PET sheet while varying the pulse beam power from 0.35 mW to 0.43 mW at the scanning speed of 3.0 μm/s and the power of CW beam of 1.79 mW. There are only some nanoparticles on the PET sheet when the power of pulse beam is 0.35 mW (Fig. 3b). But a series of AgNWs is able to be fabricated when the power is over 0.35 mW. The AgNW width increases from 134 nm to 190 nm when the pulse beam power changes from 0.36 mW to 0.39 mW, but only 8 nm at higher pulse beam power from 0.40 mW to 0.43 mW (Fig. 3e). The height vibration measured from the AFM images (Supplementary Fig. S3b) increases with the increase of the laser power (Fig. 3e), but only 9 nm at higher pulse beam power from 0.40 mW to 0.43 mW. Corresponding to the multiphoton absorption probability, the amount of Ag nanoparticles increase faster when the pulse beam power is less than 0.39 mW, but slower when the power is more than 0.40 mW. The compactness and the smoothness of the AgNWs are almost the same when the pulse beam power changes at high laser power. These results indicate that the morphology such as the width and height of AgNW is strongly dependent on the amount of Ag nanoparticles.

Differently, CW laser beam would influence the width and height of the AgNW through the optical trapping force. Here, the AgNWs were fabricated on the PET sheet while varying the laser power of CW beam from 1.55 mW to 1.79 mW at a scanning speed of 3.0 μm/s and the pulse laser power of 0.43 mW (Fig. 3c). The corresponding width and height of the AgNWs are analyzed accordingly. The widths of the AgNWs are almost constant and the roughness is less than 10 nm, and surface of the AgNWs are compact and smooth with the CW beam power ranging from 1.59 mW to 1.79 mW (Fig. 3f). The height of the AgNWs measured from the AFM image (Supplementary Fig. S3c) increases from 53 nm to 84 nm when the CW laser power changes from 1.55 mW to 1.79 mW, which is mainly attributed to the increase of the longitudinal gradient force of the CW beam induced optical trapping. Note that the height of the AgNW is the smallest and the width is the biggest when the CW laser power is 1.55 mW. In this case, the optical trapping force of CW laser beam is too weak to prompt the aggregation of Ag nanoparticles even to further improve the morphology of the AgNW. On the contrary, the volume of AgNW increases at higher CW laser power, resulting from the increase of height and the constant width. This phenomenon implies that the amount of the Ag nanoparticles increase with the increase of the CW laser power. Therefore, we found that the CW beam not only works as optical tweezers to compact the Ag nanoparticles together, but also assists the photoreduction of the Ag nanoparticles.

### The conductivity of the AgNW

**Figure 4:**
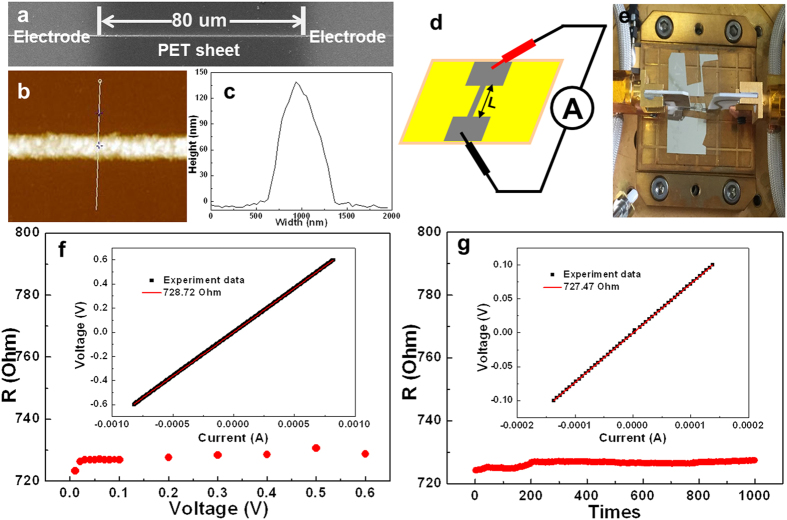
Investigated the conductivity of the AgNW. (**a**) SEM image of the AgNW between two silver electrodes. (**b**) AFM image of AgNW. (**c**) The cross-section profile of the AgNW in (**b**). (**d**) The schematic illustration of the electrical measurement. (**e**) The digital photographs of the electrical measurement setup. (**f**) The resistance variation of AgNW with the measured voltage. Inset: The I-V curve at the voltage of 0.6 V. (**g**) The resistance variation of the AgNW with the measured times. Inset: The AgNW I-V curve of the 1000^th^ time.

The conductivity is of significant importance for the metallic nanostructures because this will directly determine the promising application in electronics, photonics and so on. The resistivity of the AgNW is calculated from the equation ρ = RS/L. *R* is resistance, *S* is the cross-section area of the AgNW, and *L* is the distance of the two electrodes. The Ag nanowire for the conductivity characterization was fabricated on the PET sheet with the power of the pulse beam and the CW beam of 0.43 mW and 1.79 mW, the scanning speed of 3.0 μm/s and scanned three times. Before the resistance measurement, a metal mask plate was used to cover the individual AgNW prefabricated on the PET sheet and deposited silver electrodes on both ends. Figure 4a shows the SEM image of the AgNW and the electrodes, in which the two electrodes distance is 80 μm. The magnified AFM image of the AgNW and corresponding cross-section indicates the width and height of the AgNW (Fig. 4b,c). The schematic illustration of the electrical measurement setup and the digital photographs are shown in Fig. 4d,e, respectively.

We have measured the resistance of the AgNW under different voltages to determine the applicable voltage range. The Ag nanowire resistance corresponding to different voltage values in Fig. 4f indicates that the resistance value is not stable when the voltage value is less than 0.03 V. This is because the value of the voltage is too low and the impact of the measurement instability is relatively large. The impressed voltage instability of the measurement system is 0.03% when the voltage is less than 0.003 V. The AgNW resistivity is very stable from 0.03 V to 0.1 V, but increases with the increase of the voltage from 0.1 V to 0.5 V. The AgNW will generate Joule heat and the temperature of the AgNW must increase when the current occurs. Correspondingly, the mean freepath of the electron will get short and the AgNW resistance will increase. The higher the voltage, the faster the resistance increases. That is why the resistance value increases with the increase of the voltage value when the voltage value varies from 0.1 V to 0.5 V. It can be observed that the resistance at the voltage value of 0.6 V is smaller than that at 0.5 V, although the measured I-V curve is still linear (Inset in Fig. 4f). The resistance of the AgNW has been measured and reached to thousand even 10 thousand Ohm when the test voltage varied from 0.7 V to 1 V (Supplementary Fig. S4). This indicates that the structure of AgNW has greatly changed due to the Joule heat of electric current. At the same time, the melting point of the nanoparticle will decrease sharply if the nanoparticle size is less than 40 nm^38^. The AgNW temperature may be higher than its melting point and the AgNW will be fused when the voltage is bigger than 1 V. Therefore, the voltage changed range for the most stable AgNW resistance is from 0.03 V to 0.1 V, and the corresponding voltage per meter range is from 3.75 × 10^2^ to 1.25 × 10^3^ V/m. The applicable voltage per meter range is determined to be less than 7.5 × 10^3^ V/m for the fabricated AgNW.

The AgNW resistance stability is important regarding to the practical applications. Figure 4g shows the AgNW resistance dependence on the times of the current flow. The data has no big change although the resistance was measured 1000 times in the matter of one hour. Among these results, the biggest change of AgNW is only 0.44%. The I-V curve for AgNW of the 1000^th^ measured times was still of good linear relationship (inset of Fig. 4g). This implies that the stability of AgNW resistance is very excellent, which is important for the potential applications in electronic or photonics.

The AgNW resistivity is (4.583 ± 0.305) × 10^−7^ Ohm·m, which is larger than the bulk Ag resistivity (1.587 × 10^−8^ Ohm·m). We use the NDSS solution as the surfactant, the residual molecules of the NDSS decrease the conductivity significantly^12^,^13^. Generally, scattering at the surface and reflection at grain boundary of conduction electron may influence the resistance of the nanowire if the scales of nanowire are comparable to the electron mean freepath^39^,^40^. The electron mean freepath of bulk Ag is about 52 nm^41^ at the temperature of 295 K. The height and the full width at the half maximum of the AgNW are 141 ± 15 nm and 480 ± 7 nm (Fig. 4b,c), which are all larger than the mean freepath of bulk Ag. In the regime where the width of the wire is a few tens of nanometers, it has been established adequately that the resistivity is not only determined by the material but its size as well^42^,^43^. For nanowires with diameters approaching molecular dimensions the transport is likely to be quantum in nature^44^. The AgNW that fabricated by two-beam laser method is composed of Ag nanoparticles with the size of about 20 nm^25^. The electron free path range of a AgNW with a radius of 100 ± 50 nm is from 21 nm to 36 nm at the temperature of 295 K^45^. The conduction electrons scattering at the Ag nanoparticles surface are the dominant contribution to the electric resistance of the AgNW. Due to the roughness of the AgNW surface, the conduction electrons scattering at the AgNW surface could not be neglected and would lead to the increase of the resistivity.

### Transport in AgNW on flexible sheet

**Figure 5:**
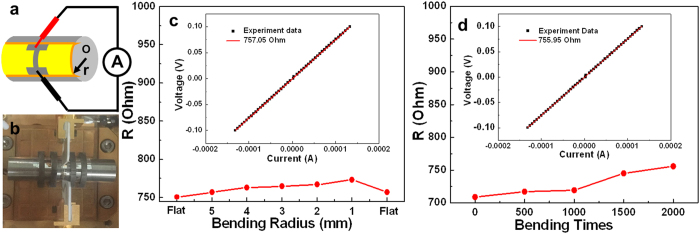
Investigated the flexibility of the AgNW. (**a**) The schematic illustration of the resistance measurement setup under bending deformation. (**b**) The digital photograph of the resistance measurement setup under bending deformation. (**c**) The AgNW resistance variation with the different bending radii, the “Flat” on the right side and left side of the coordinate represent without bending and straightening after bending, respectively. Inset: The AgNW I-V curve when it was straightened. (**d**) The resistance variation of AgNW with the bending times. Inset: The AgNW I-V curve after it was stretched bending 2000 times.

The AgNW that fabricated on the PET sheet is of good prospect for the application in the flexible electronics. To prove the flexibility, the other AgNW was used to investigate the resistance variation under bending deformations (Supplementary Fig. S5). Figure 5a,b are the schematic illustration and the digital photograph of the resistance characterization setup under bending deformation. The resistance displays a slight increase with the decrease of the bending radius, and can almost recover after bending (Fig. 5c). Here, the resistance of the AgNW without bending is 750.69 Ohm, the resistance of the AgNW after bending increases 6.36 Ohm and the I-V curve is of linear (Fig. 5c inset). The relative increase rate is only 0.85%. This indicates the particle-particle intervals^12^ inside the nanowire would increase when the bending radius gets smaller, and the particle-particle intervals can recover when it is straightened. Notably, the AgNW resistance increases 22.59 Ohm and the I-V curve is still linear at a bending radius of 1 mm as well as at other bending radii (Supplementary Fig. S5), and the increase rate is only 3.10% relative to that without bending. This result implies that the structure of AgNW is very compact, and the separated distance between the Ag nanoparticles of the AgNW is too short to destroy the AgNW inner structure when it is bended even at a small bending radius of 1 mm.

To further display the flexibility of the AgNW on the PET sheet, the resistance changes with the bending cycles have been further investigated. The AgNW still shows a good conductivity with an electrical resistance increasing from 708.92 Ohm to 755.95 Ohm even after 2000 times bending cycles at the bending radius of 1 mm (Fig. 5d), with the increase of 6.63%. In addition, as well as all of the other I-V curves of the AgNW corresponding to the bending cycles (Supplementary Fig. S6), the I-V curve of the AgNW that after 2000 bending cycle times at the bending radius of 1 mm is also linear. It reveals that the conductance of the AgNW is hardly affected by the bending stress, and indicating well ohm contact among Ag nanoparticles inside the AgNW. We believe the CW beam in this study not only contributes to the compact Ag nanoparticles in the AgNW because of CW laser induced optical trapping force, but also helps the adherence of AgNW to PET substrate because of the CW laser thermal effect. These results indicate the excellent electromechanical stability of AgNW and the great potential for high-performance flexible electronics.

## Conclusion

In conclusion, we have successfully fabricated the AgNW on PET sheet by two-beam laser direct writing technique and investigated the conductivity and flexibility. We can monitor the morphology of the AgNW by changing the experimental conditions, and the minimum width of the AgNW can reach to 134 nm with a height of 49 nm. The applicable voltage per meter range of AgNW is less than 7.5 × 10^3^ V/m. The maximum change of the resistivity is only about 0.44% even after 1000 times measurement in the matter of one hour. Compared to the AgNW resistance without bending, the resistance is only increased 3.10% when the AgNW is bended to the radius of 1 mm, and the increase is only 0.85% when the AgNW is flat again. Furthermore, the resistance variation of the AgNW is only 6.63% even after stretched bending 2000 times at such a small bending radius of 1.0 mm. The proposed protocol would provide high potential for the fabrication of conductive metal nanowires on the flexible substrate, which would benefit for the flexible electronics.

## Methods

### Materials preparation

AgNO_3_ and NDSS were purchased from Alfa Aesar (China) Chemicals Co., Ltd and Tokyo Chemical Industry Co., Ltd, respectively. Materials for photoreduction were prepared according to the literature^25^. Typically, 0.0340 g of AgNO_3_ was dissolved in 1.8 mL of water, and 0.2 mL of ammonia (25 wt %) water was added, so the solution of diammine silver ions (DSI) was formed. Then, 0.0525 g of nitrogen-atom containing alkyl carboxylate (n-decanoylsarcosine sodium, NDSS) was dissolved in 1 mL water and mixed with 1 mL diammine silver ions solution. The Ag aqueous solution consists of 1 mL of DSI solution (0.05 M) and 1 mL of NDSS solution (0.099 M) as surfactant.

### Characterization

The size and shape of the Ag nanowire were investigated by scanning electron microscopy (SEM, QUANTA FEG 250 USA), at an accelerating voltage of 10.0 kV. The height of the Ag nanowire was characterized by AFM measurement (Bruker Multimode 8) at a scanning rate of 0.8 Hz in dynamic contact mode.

### Electrical measurement

The electrical measurement was characterized under nitrogen environment conditions, using a Keilthley 4200-SCS semiconductor system and the CRX-4K Cryogenic probe station in a clean and shielded box in the dark condition. Two silver electrodes were deposited on the both ends of the Ag nanowire by radio frequency magnetron sputtering (QPrep 400-BASE). Two golden conical probes were placed to contact with two deposited electrodes, then applying impressed voltage to detect the corresponding current. The electrical measurement for flexible AgNW on PET sheet was operated by using a home-made bending equipment as shown in Fig. 5a,b.

## Additional Information

**How to cite this article**: He, G.-C. *et al*. The Conductive Silver Nanowires Fabricated by Two-beam Laser Direct Writing on the Flexible Sheet. *Sci. Rep.* **7**, 41757; doi: 10.1038/srep41757 (2017).

**Publisher's note:** Springer Nature remains neutral with regard to jurisdictional claims in published maps and institutional affiliations.

## Supplementary Material

Supplementary Information

## References

[b1] Li T., Lee J. & Akinwande D. Nanofabrication down to 10 nm on a plastic substrate. J. Vac. Sci. Technol. B 29, 06FG07 (2011).

[b2] Han J., Choi S., Lim J., Lee B. S. & Kang S. Fabrication of transparent conductive tracks and patterns on flexible substrate using a continuous UV roll imprint lithography. J. Phys. D: Appl. Phys. 42, 115503 (2009).

[b3] Rogers J. A., Someya T. & Huang Y. Materials and mechanics for stretchable electronics. Science 327, 1603–1607 (2010). 20339064 10.1126/science.1182383

[b4] Hong S. . Highly stretchable and transparent metal nanowire heater for wearable electronics applications. Adv. Mater. 27, 4744–4751 (2015). 26177729 10.1002/adma.201500917

[b5] Deng X., Koopmanb M., Chawla N. & Chawla K. K. Young’s modulus of (Cu, Ag)–Sn intermetallics measured by nanoindentation. Materials Science and Engineering. Mater. Sci. Eng. A 364, 240–243 (2004).

[b6] Kaneko K., Sun H. B., Duan X. M. & Kawata S. Two-photon photoreduction of metallic nanoparticle gratings in a polymer Matrix. Appl. Phys. Lett. 83, 1426–1428 (2003).

[b7] Ishikawa A., Tanaka T. & Kawata S. Improvement in the reduction of silver ions in aqueous solution using two-photon sensitive dye. Appl. Phys. Lett. 89, 113102 (2006).

[b8] Tanaka T., Ishikawa A. & Kawata S. Two-photon-induced reduction of metal ions for fabricating three-dimensional electrically conductive metallic microstructure. Appl. Phys. Lett. 88, 081107 (2006).

[b9] Baldacchini T. . Multiphoton laser direct writing of two dimensional silver structures. Opt. Express 13, 1275–1280 (2005). 19495000 10.1364/opex.13.001275

[b10] Son Y., Lim T. W. & Yang D. Y. Improvement of two-photon induced photoreduction by using a metal ion solution with a high concentration of silver ions. Int. J. Nanomanufacturing 6, 219–230 (2010).

[b11] Zhang Z. . Controlled inkjetting of a conductive pattern of silver nanoparticles based on the coffee-ring effect. Adv. Mater. 25, 6714–6718 (2013). 24123367 10.1002/adma.201303278

[b12] Xu B. B. . Flexible nanowiring of metal on nonplanar substrates by femtosecond-laser-induced electroless plating. Small 6, 1762–1766 (2010). 20665756 10.1002/smll.201000511

[b13] Xu B. B. . Fabrication of microelectrodes based on precursor doped with metal seeds by femtosecond laser direct writing. Opt. Lett. 39, 434–437 (2014). 24487833 10.1364/OL.39.000434

[b14] Wang X., Wang R., Shi L. & Sun J. Synthesis of metal/bimetal nanowires and their applications as flexible transparent electrodes. Small 11, 4737–4744 (2015). 26179912 10.1002/smll.201501314

[b15] Xia X., Wang Y., Ruditskiy A. & Xia Y. 25th Anniversary Article: Galvanic replacement: a simple and versatile route to hollow nanostructures with tunable and well-controlled properties. Adv. Mater. 25, 6313–6333 (2013). 24027074 10.1002/adma.201302820

[b16] Jin M. . Shape-controlled synthesis of copper nanocrystals in an aqueous solution with glucose as a reducing agent and hexadecylamine as a capping agent. Angew. Chem. Int. Ed. 50, 10560–10564 (2011). 10.1002/anie.201105539 21928444

[b17] Guo H. Z., Chen Y., Ping H., Jin J. & Peng D. L. Facile synthesis of Cu and Cu@Cu–Ni nanocubes and nanowires in hydrophobic solution in the presence of nickel and chloride ions. Nanoscale 5, 2394–2402 (2013). 23400550 10.1039/c3nr33142c

[b18] Korte K. E., Skrabalak S. E. & Xia Y. Rapid synthesis of silver nanowires through a CuCl- or CuCl_2_-mediated polyol process. J. Mater. Chem. 18, 437–441 (2008).

[b19] Gao H. L. . Rapid synthesis of silver nanowires through a CuCl- or CuCl_2_-mediated polyol process. Angew. Chem. Int. Ed. 53, 4561–4566 (2014).

[b20] Liu J. W. . Manipulating nanowire assembly for flexible transparent electrodes. Angew. Chem. Int. Ed. 53, 13477–13482 (2014). 10.1002/anie.201408298 25283948

[b21] Banu S. . Fabrication of diffraction-encoded micro-particles using nano-imprint lithography, diffraction patterns. J. Micromech. Microeng. 17, S116–S121 (2007).

[b22] Singh M., Haverinen H. M., Dhagat P. & Jabbour G. E. Inkjet printing—process and its applications, Adv. Mater. 22, 673–685 (2010). 20217769 10.1002/adma.200901141

[b23] Chen S. . Fabrication of nanoscale circuits on inkjet-printing patterned substrates. Adv. Mater. 27, 3928–3933 (2015). 26011403 10.1002/adma.201500225

[b24] Jiang J. . Fabrication of Transparent multilayer circuits by inkjet printing. Adv. Mater. 28, 1420–1426 (2016). 26643356 10.1002/adma.201503682

[b25] Cao Y. Y., Takeyasu N., Tanaka T., Duan X. M. & Kawata S. 3D metallic nanostructure fabrication by surfactant-assisted multiphoton-induced reduction. Small 5, 1144–1148 (2009). 19291732 10.1002/smll.200801179

[b26] Cao Y. Y. . Morphology and size dependence of silver microstructures in fatty salts-assisted multiphoton photoreduction microfabrication. Appl. Phys. A 96, 453–458 (2009).

[b27] Jin W. . Morphology modification of silver microstructures fabricated by multiphoton photoreduction. J. Nanosci. Nanotechnol. 11, 8556–8560 (2011). 22400224 10.1166/jnn.2011.4971

[b28] Wang W. K. . Magnetic nickel-phosphorus/polymer composite and remotely driven three-dimensional micromachine fabricated by nanoplating and two-photon polymerization. J. Phys. Chem. C 115, 11275–11281 (2011).

[b29] Lu W. E. . Femtosecond direct laser writing of gold nanostructures by ionic liquid assisted multiphoton photoreduction. Opt. Mater. Express 3, 1660–1673 (2013).

[b30] Jia Y. P. . Complementary chiral metasurface with strong broadband optical activity and enhanced transmission. Appl. Phys. Lett. 104, 011108 (2014).

[b31] Zhao Y. Y. . Tailored silver grid as transparent electrodes directly written by femtosecond laser. Appl. Phys. Lett. 108, 221104 (2016).

[b32] Novotny L., Bian R. X. & Xie X. S. Theory of nanometric optical tweezers, Phys. Rev. Lett. 79, 645–648 (1997).

[b33] Yan Z., Sajjan M. & Scherer N. F. Fabrication of a material assembly of silver nanoparticles using the phase gradients of optical tweezers. Phys. Rev. Lett. 114, 143901 (2015). 25910124 10.1103/PhysRevLett.114.143901

[b34] Ashkin A. Acceleration and trapping of particles by radiation pressure, Physical review letters. Phys. Rev. Lett. 24, 156–159 1970.

[b35] Ashkin A., Dziedzic J. M., Bjorkholm J. E. & Chu S. Observation of a single-beam gradient force optical trap for dielectric particles. Opt. Lett. 11, 288–290 (1986). 19730608 10.1364/ol.11.000288

[b36] Svoboda K. & Block S. M. Optical trapping of metallic Rayleigh particles. Opt. Lett. 19, 930–932 (1994). 19844491 10.1364/ol.19.000930

[b37] Barton J. P. & Alexander D. R. Fifth-order corrected electromagnetic field components for a fundamental Gaussian beam. Optical trapping of metallic Rayleigh particles. J. Appl. Phys. 66, 2800–2802 (1989).

[b38] Buffat P. & Borel J. P. Size effect on the melting temperature of gold particles. Phys. Rev. A 13, 2287–2298 (1976).

[b39] Sondheimer E. H. The mean free path of electrons in metals. Adv. Phys. 1, 1–42 (1952).

[b40] Mayadas A. F. & Shatzkes M. Electrical-resistivity model for polycrystalline films: the case of arbitrary refection at external surfaces. Phys. Rev. B 1, 1382–1389 (1970).

[b41] Brioude A. & Pileni M. P. Silver nanodisks: Optical properties study using the discrete dipole approximation method. J. Phys. Chem. B 109, 23371–23377 (2005). 16375309 10.1021/jp055265k

[b42] Steinhög W., Schindler G., Steinlesberger G. & Engelhardt M. Size-dependent resistivity of metallic wires in the mesoscopic range. Phys. Rev. B 66, 075414 (2002).

[b43] Wu W., Brongersma S. H., Van Hove M. & Maex K. Influence of surface and grain-boundary scattering on the resistivity of copper in reduced dimensions. K. Appl. Phys. Lett. 84, 2838 (2004).

[b44] Imry Y. Introduction to Mesoscopic Physics (ed. Craighead H. G. .) 12–34 (New York, 1997).

[b45] Bid A., Bora A. & Raychaudhuri A. K. Temperature dependence of the resistance of metallic nanowires of diameter ≥15 nm: Applicability of Bloch-Grüneisen theorem. Phys. Rev. B 74, 035426 (2006).

