# Supplementary material for: The Conductive Silver Nanowires Fabricated by Two-beam Laser Direct Writing on the Flexible Sheet
Source: Sci Rep. 2017 Feb 2;7:41757. doi: 10.1038/srep41757 (PMC5288690; doi:10.1038/srep41757)
Supplement: Supplementary Information [file srep41757-s1.pdf]

Supplementary Information

The Conductive Silver Nanowires Fabricated by Two-beam Laser Direct Writing on the Flexible Sheet

Gui-Cang He[1,3], Mei-Ling Zheng[1,*], Xian-Zi Dong[1], Feng Jin[1], Jie Liu[1], Xuan-Ming Duan[2,*], and Zhen-Sheng Zhao[1]

[1]Laboratory of Organic NanoPhotonics and Laboratory of Bio-Inspired Smart Interface Science, Technical Institute of Physics and Chemistry, Chinese Academy of Sciences, No. 29, Zhongguancun East Road, Beijing, 100190, P. R. China
[2]Chongqing Institute of Green and Intelligent Technology, Chinese Academy of Sciences, No.266 Fangzheng Ave, Shuitu technology development zone, Beibei District, Chongqing 400714, P. R. China
[3]University of Chinese Academy of Sciences, No.29, Zhongguancun East Road, Beijing, 100190, P. R. China
[*]zhengmeiling@mail.ipc.ac.cn; xmduan@cigit.ac.cn.

1. Fabricated AgNWs on PET sheet by femtosecond pulse laser

The AgNWs have been fabricated on PET sheet by only one femtosecond pulse laser with a center wavelength of 780 nm. The laser power was kept at 0.85 mW, and the AgNWs were fabricated on the PET sheet by varying laser scanning speed from 1.0 μm/s to 3.5 μm/s. As shown in Fig. S1a, a very loosely continuous AgNW is fabricated on the PET sheet when the laser focus scanning speed is 1.0 μm/s, and others are only some discontinuous Ag nanoparticles. Then the laser focus scanning speed is kept at 1.0 μm/s, and the AgNWs are fabricated on the PET sheet while varying power of pulse laser from 0.63 mW to 0.85 mW. We can see in Fig. S1b that a very loosely continuous AgNW was fabricated on the PET sheet when the pulse laser power is 0.85 mW, and others are only some discontinuous Ag nanoparticles. That indicates a compact continuous AgNW could not be fabricated on PET sheet by only one femtosecond pulse laser.

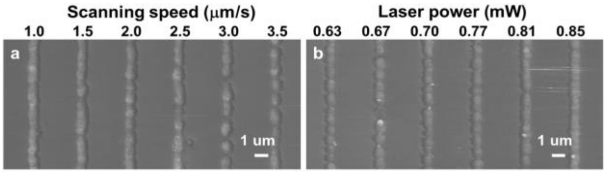

Figure S1. SEM images of the AgNWs on the PET sheet. a) SEM image of AgNWs fabricated while varying the laser focus scanning speed from 1.0 μm/s to 3.5 μm/s. The power of femtosecond pulse laser is 0.85 mW. **b)** SEM image of AgNWs fabricated while the femtosecond pulse power varied from 0.63 mW to 0.85 mW. The laser focus scanning speed is 1.0 μm/s.

2. Fabricated AgNWs on PET sheet by CW laser

AgNWs have been fabricated on PET sheet by a CW laser with a wavelength of 442 nm. The laser power was kept at 2.47 mW, and the AgNWs were fabricated on the PET sheet while varying the laser focus scanning speed from 1.0 μm/s to 3.5 μm/s. As shown in Fig. S2a, only some Ag nanoparticles were fabricated on PET sheet, and some big Ag nanoparticles were generated when the scanning speed was more than 2.0 μm/s. Then the laser focus scanning speed was kept at 1.0 μm/s, and the AgNWs were fabricated on the PET sheet while varying power of CW laser from 1.49 mW to 3.30 mW. In Fig. S2b, some Ag nanoparticles were fabricated on PET sheet, and some big Ag nanoparticles appeared when the laser power was more than 2.10 mW. Thus a compact continuous AgNW could not be fabricated on PET sheet by only CW laser, but the CW laser photoreduction effect of the Ag nanoparticles are not ignored under certain experimental condition.

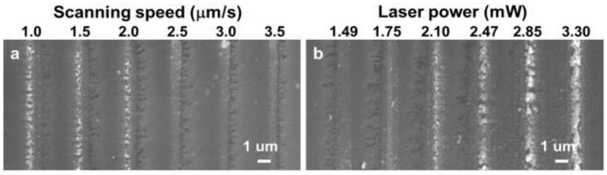

Figure S2. SEM images the AgNWs on the PET sheet. a) SEM image of AgNWs fabricated by varying the laser scanning speed from 1.0 μm/s to 3.5 μm/s. The power of CW laser is 2.47 mW. **b)** SEM image of AgNWs fabricated with the CW laser power varied from 1.49 mW to 3.30 mW. The laser scanning speed is 1.0 μm/s.

3. The AFM image of the AgNW fabricated by two-beam laser technique

The height and the cross section of AgNW can be obtained from the AFM image. Fig. S3a is the AFM image of the AgNWs, which were fabricated with different scanning speed while the laser power for the pulse beam and the CW beam were kept at 0.43 mW and 1.79 mW. The AgNW height (Fig. S3d) and cross section area were decreased with the increasing of the laser scanning speed, indicating the amount of the Ag nanoparticles decrease. Fig. S3b is the AFM image of the AgNW fabricated with different power of pulse laser beam, while the laser power for the CW beam and the scanning speed are kept at 1.79 mW and 3.0 μm/s. As shown in Fig. S3e, the height of AgNWs increased when pulse laser power changed from 0.35 mW to 0.43 mW. Besides, the cross section of AgNW also changed when the pulse laser power changed from 0.35 mW to 0.39 mW, but it was almost unchanged when the pulse laser power changed from 0.40 mW to 0.43 mW. This indicates that multiphoton absorption probability increased with the increasing of the pulse beam power when the pulse beam power changes from 0.35 mW to 0.39 mW, but the increasing rate of multiphoton absorption probability is very little or there are not enough silver ions to be reduced into silver atoms when the pulse beam power changes from 0.40 mW to 0.43 mW. Fig. S3c is the AFM image of the AgNW, which were fabricated with different power of CW laser beam, while the laser power for the pulse beam and the scanning speed are kept at 0.43 mW and 3.0 μm/s. As shown in Fig. S3c,f, both the cross section area and the height of AgNWs increased when CW laser power varies from 1.55 mW to 1.79 mW. Therefore, we believe that the CW beam not only works as optical tweezers to compact the Ag nanoparticles together, but also assists the photoreduction of the Ag nanoparticles.

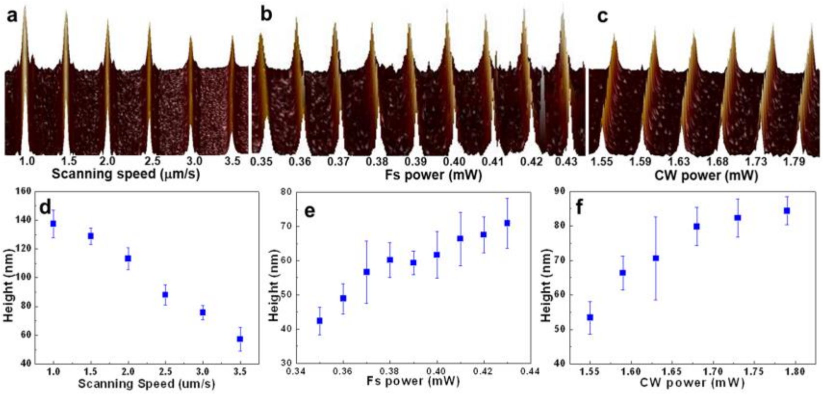

Figure S3. AFM images and the corresponding height of the AgNWs on the PET sheet. a) AFM image of AgNWs fabricated by varying the scanning speed from 1.0 μm/s to 3.5 μm/s. The laser powers for pulse beam and CW beam are 1.79 mW and 0.43 mW, respectively. **b)** AFM images of the AgNWs fabricated while the pulse beam power varied from 0.35 mW to 0.43 mW. The CW beam power and scanning speed are 1.79 mW and 3.0 μm/s. **c)** AFM image of AgNWs fabricated while the CW beam power varied from 1.55 mW to 1.79 mW. The pulse beam power is 0.43 mW and the scanning speed is 3.0 μm/s. **d)** The dependence of height of the AgNWs in (a) on the scanning speed. **e)** The dependence of height of the AgNWs in (b) on the pulse beam power.

f) The dependence of height of the AgNWs in (c) on the CW beam power.

4. The AgNW resistance tested under different measured voltages

The AgNW resistance was measured under different measurement voltages. As shown in Fig. S4, the resistance value of AgNW is almost unchanged with the change of the measured voltage value less than 0.6 V. But the resistance value increases sharply when the measured voltages from 0.7 V to 1 V. This indicates that the structure of AgNW has changed greatly by the thermal effect of electric current when measured voltage changed from 0.7 V to 1 V.

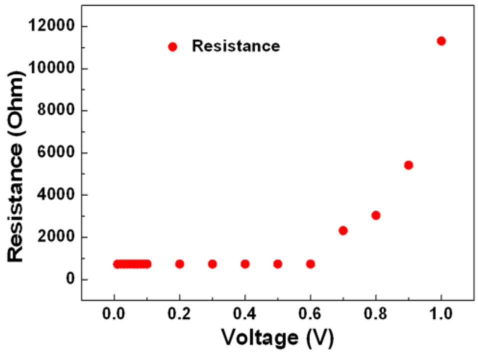

Figure S4. The resistance variation of AgNW with the measured voltage.

5. The AgNW resistance measured under different bending radii

The AgNW resistance was measured under different bending radii. Fig. S5a shows the SEM image of the AgNW and the electrodes. Fig. S5b,c show the image of the AgNW and its cross-section measured by AFM. Fig. S5d-I are I-V curves of the AgNW under deferent bending conditions. All the I-V curves shown in Fig. S5 are of good linear. This result implies the separated distance between the Ag nanoparticles of the AgNW are too short to destroy the AgNW inner structure and Ag nanoparticles inside the AgNW well ohm contact when it was bended at different bending radii.

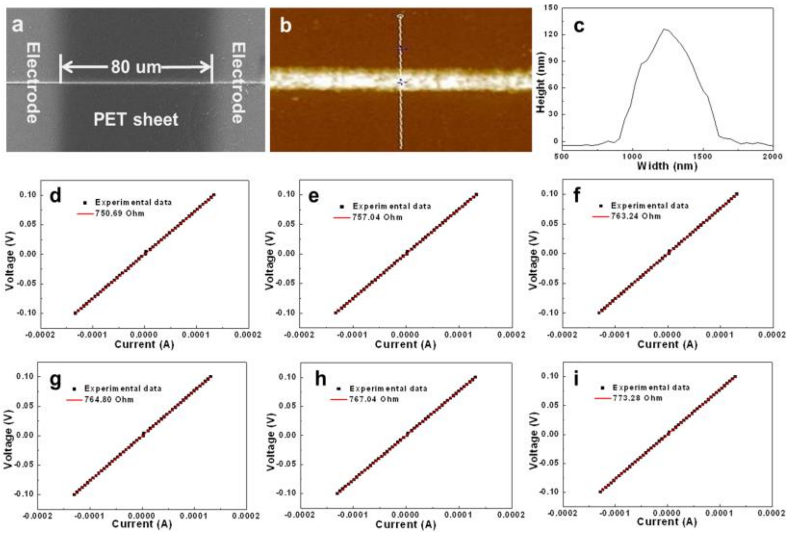

Figure S5. The AgNW resistance measured under different bending radii. a) SEM image of the AgNW between two silver electrodes. **b)** AFM image of AgNW. **c)** The cross-section profile of the AgNW in (b). **d)** The I-V curve of the AgNW without bending. **e)** The AgNW I-V curve at the bending radius of 5 mm. **f)** The AgNW I-V curve at the bending radius of 4 mm. **g)** The AgNW I-V curve at the bending radius of 3 mm. **h)** The AgNW I-V curve at the bending radius of 2 mm. **i)** The AgNW I-V curve at the bending radius of 1 mm.

6. The AgNW resistance measured under different bending times

The AgNW resistance was measured under different bending times at the bending radius of 1mm. Fig. S6a shows the SEM image of the AgNW and the electrodes. Fig. S6b,c show the AFM image of the AgNW and the corresponding cross-section. Fig. S6d-g are the I-V curves of the AgNW under deferent bending times at the bending radius of 1 mm. The four I-V curves shown in Fig. S6 are all of linear. It reveals that the conductance of the AgNW is hardly affected by bending stress, and indicating good ohm contact among Ag nanoparticles inside the AgNW after bending different times at the bending radius of 1 mm.

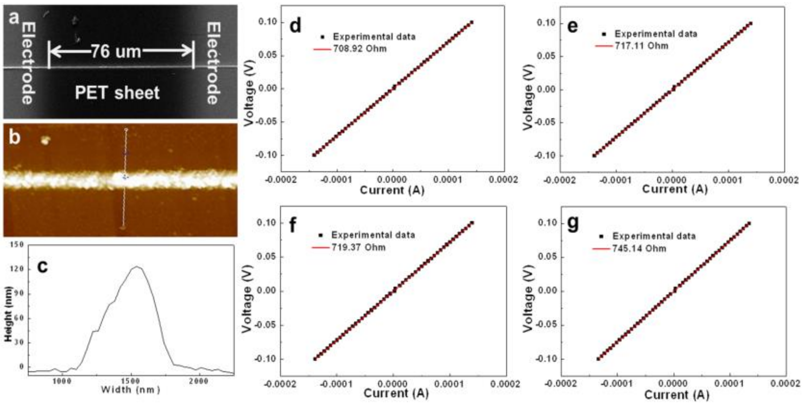

Figure S6. The AgNW resistance measured under different bending times. a) SEM image of the AgNW between two silver electrodes. **b)** AFM image of AgNW. **c)** The cross-section profile of the AgNW in (b). **d)** The I-V curve of the AgNW without bending. **e)** The I-V curve of the AgNW with the bending times of 500 at the bending radius of 1 mm. **f)** The I-V curve of the AgNW with the bending times of 1000 at the bending radius of 1 mm. **g)** The I-V curve of the AgNW with the bending times of 1500 at the bending radius of 1 mm.
